# An Experimental Investigation to Use the Biodiesel Resulting from Recycled Sunflower Oil, and Sunflower Oil with Palm Oil as Fuels for Aviation Turbo-Engines

**DOI:** 10.3390/ijerph18105189

**Published:** 2021-05-13

**Authors:** Grigore Cican, Marius Deaconu, Radu Mirea, Laurentiu Constantin Ceatra, Mihaiella Cretu

**Affiliations:** 1 Faculty of Aerospace Engineering, Polytechnic University of Bucharest, 1-7 Polizu Street, 1, 011061 Bucharest, Romania; 2 National Research and Development Institute for Gas Turbines COMOTI, 220D Iuliu Maniu, 061126 Bucharest, Romania; marius.deaconu@comoti.ro (M.D.); radu.mirea@comoti.ro (R.M.); laurentiu.ceatra@comoti.ro (L.C.C.); mihaela.cretu@comoti.ro (M.C.)

**Keywords:** biodiesel, aviation, recycle, sunflower oil, sunflower plus palm oil, fuel

## Abstract

The paper is presenting the experimental analysis of the use of biodiesel from waste sunflower oil and a blend of sunflower oil with palm oil as fuel for aviation turbo-engines. A comparative analysis for fuel mixtures made of Jet A + 5% Aeroshell 500 Oil (Ke) with 10%, 30%, and 50% for each bio-fuel type has been performed and Ke has been used as reference. Firstly, the following physical and chemical properties were determined: density, viscosity, flash point, freezing point, calorific power. Then, elemental analysis and Fourier transform infrared spectroscopy (FTIR) analysis were conducted for Ke, biodiesel obtained from recycled sunflower oil (SF), biodiesel obtained from blending recycled sunflower oil, and recycled palm oil (SFP), and for each fuel blend. Secondly, experimental tests of the blends have been conducted on the Jet Cat P80^®^ micro-turbo engine (Gunt Hamburg, Barsbüttel, Germany). The tests have been conducted at different engine working regimes as follows: idle, cruise, intermediate, and maximum. For each regime, a one-minute testing period was chosen, and the engine parameters have been monitored. The turbo engine instrumentation recorded the temperature after the compressor and before the turbine, the fuel consumption and air flow, pressure inside the combustion chamber, and generated thrust. The burning efficiency and the specific consumption have been calculated for all four above-mentioned regimes and for all fuel blends. Two accelerometers have been installed on the engine’s support to register radial and axial vibrations allowing the assessment of engine stability.

## 1. Introduction

Nowadays, it is known that the problems related to the emission of pollutants and greenhouse gases resulted from the fossil fuels burning and the problem of waste have a serious effect on the environment and on the quality of life of the people [1].

Globally, there are many sources of pollution with environmental effects, one of the most important polluting sectors being the transport, and a fair share of it is represented by aviation [2,3].

The Aviation Researcher group has tried to mitigate the environmental issues generated by the emissions, and a considerable decrease by up to 75% for CO_2_ and up to 90% for NO_x_ emissions/passenger x km has been envisioned in “Flightpath 2050” considering the technologies and procedures that will be available by then [4]. According to the European Aviation Environmental Report, by 2040, CO_2_ and NO_x_ emissions are predicted to increase by at least 21% and 16%, respectively [5].

The International Civil Aviation Organization’s (ICAO) 2050 Vision for Sustainable Aviation Fuels states that unlike other sectors (e.g., road transport), which can apply to different energy sources, the air transport must have a significant proportion of conventional aviation fuels to be substituted with sustainable aviation fuels by 2050 [6].

The experts highlight that natural resources such oil and gas will end completely if the same consumption pattern is kept for the next 60 years [7]. Thus, the most feasible alternative sources of fuel for turbo-engines are the bio-fuels [8].

There are several ways and procedures to obtain biodiesel [9]. It can be produced from the following raw materials: vegetable oils, animal fat, waste oil/fat, algae, thus resulting biodiesel, crude bio-oil, bio ethanol, biogas, and bio hydrogen with the amendment that, in general, liquid fuels are used for conventional engine infrastructure. In order to successfully convert the feedstock into an actually usable bio-fuel for turbo-engines, several conversion paths are available: gas to jet, oil to jet, sugar to jet, and alcohol to jet [10,11,12]. Even though the above-mentioned technologies are widely used in the industrial sector, the most important one is represented by oil-to-jet, which implies hydro processing [13]. In paper [14], the way to produce biodiesel from fat and waste oil was investigated, and also an evaluation of spirulina, waste cooking oil, and animal fats usage for biodiesel production was assessed within the paper [15]. Perspectives and challenges regarding the use of biomass-derived aviation fuels are presented in [16,17].

Bio-fuels are used in many engineering fields where applications involve thermal engines and more precisely the internal combustion engines. Thus, some research studies focused on the use of bio-fuel/biodiesel to feed internal combustion engines are presented in [18,19].

Several studies have been conducted aiming to assess the use of alternative fuels in turbo engines, consisting of both in lab and in-flight assessment [20,21,22]. Other studies that are evaluating the combustion and gaseous emissions of bio-jet fuel blends in a gas turbine combustor are described in [23,24,25,26].

In previous years, demonstration flights have been conducted in order to test the viability of drop-in fuel in real working conditions. Aircraft types as Boeing 787, 737–800, 747–400, Bombardier Q400, Airbus A321, and Falcon 20 have undertaken demonstrative flights, the aircrafts being powered with different combustible blends comprising of Jet A fuel and bio-fuel during the tests [27,28,29,30].

In addition to their importance in food manufacturing, facing the imminent ending of the conventional fuels, vegetable oils and animal fat have been envisaged as valuable renewable resources for biodiesel manufacturing.

The continuous generation of waste is creating waste management problems since the use of traditional waste management methods, such as incineration and landfill, releases greenhouse gases with a significant impact on global warming.

The waste represented by the used sunflower or palm oil and their mixture respectively can be used as a viable source for bio-fuel production, thereby solving two problems: decrease soil and water pollution and fuel production from regenerative wastes [31,32,33].

The paper is exploring the possibility of using biodiesel produced from recycled sunflower oil and a mixture of sunflower and palm oil as fuel for aviation turbo-engines.

## 2. Experimental Assessment of Fuel Blends Properties—Equipment and Testing Methods

This section includes a comparative analysis for fuel blends consisting of (1) Jet A fuel +5% Aeroshell 500 Oil (Ke), (2) biodiesel prepared from recycled sunflower oil (SF), (3) biodiesel prepared from mixing recycled sunflower oil and recycled palm oil (SFP), (4) Ke + 10% SF, (5) Ke + 30% SF, (6) Ke + 50% SF, (7) Ke + 10% SFP, (8) Ke + 30% SFP, and (9) Ke + 50% SFP.

The studied biodiesels were obtained with the transesterification production process.

The physical–chemical properties were determined for each blend followed by tests performed the Jet Cat P80^®^ micro-turbo engine using these blends as fuel.

Equipment and the testing methods used to determine Ke, SF, SFP, and the above-mentioned mixtures are presented in the following paragraphs.

### 2.1. Density Determination of the Fuel Blends

The density of all 9 above-mentioned liquids was experimentally determined by the means of the thermo-densimeter instruments. The experiments were performed as in SR EN ISO 3675/2002 [34].

### 2.2. Flash Point Determination

The Flash Point of a fuel/fuel blend is the lowest temperature at which vapors above a volatile combustible substance ignite in air when these are exposed to flame.

The Flash Point of all 9 above-mentioned liquids was experimentally determined using Automatic Flash Point Tester equipment, produced by Scavini, Banevo, Italy. The experiments were performed based on the ASTM D92 [35] test method.

### 2.3. Kinematic Viscosity Calculation

The kinematic viscosity at 40 °C was experimentally determined using a capillary viscometer immersed in a thermostatic bath having a mechanical stirrer and temperature control device. The necessary time to flow through a standardized capillary tube, for a known volume of sample was measured, and kinematic viscosity is calculated by multiplying the measured time (in seconds) with the capillary constant. The measurement unit is mm^2^/s (1 mm^2^/s = 1 cSt).

The experiments to determine the kinematic viscosity (at 40 °C) were performed according to SR EN ISO 3104/2002 [36]

### 2.4. Calorific Power Calculation

The calorific power was experimentally determined by using an IKA WERKE C 2000 isothermal calorimeter equipped with C 5012 calorimeter bomb manufactured by IKA Analisentechnik GmbH, Staufen, Germany.

The experiments for the calorific power value were performed in accordance with ASTM D240-17 [37] “Standard test method for Heat of Combustion of Liquid Hydrocarbon Fuels by Bomb Calorimeter”.

### 2.5. Freezing Point Calculation

The experimental procedure for determination of the freezing point respects the requirements specified in SR 13552:2012 [38].

The freezing point was experimentally determined by the means of a Freezing Point Analyzer produced by GGT, Giovanni Giaccardo—Torino, Italy.

It is to be mentioned that the equipment allows the determination of a freezing point as low as −35 °C.

### 2.6. FTIR Analysis (Fourier Transform Infrared Spectroscopy)

The FTIR for all samples was experimentally determined using a Spectrum OilExpress Series 100, v 3.0 spectrometer (PerkinElmer Life and Analytical Sciences, Beaconsfield, UK) for all fuels blends.

### 2.7. Elemental Analysis

A CHN-O elemental analysis was performed for estimating these components for the Ke, (SF), and (SFP), respectively all tested fuel blends.

The percentage of carbon, hydrogen, nitrogen, and oxygen content obtained by the means of this analysis are tabulated further on.

The procedure for determination of the elemental analysis meets the requirements of the standard ASTM D 5291–16: “Standard Test Methods for Instrumental Determination of Carbon, Hydrogen and Nitrogen in Petroleum Products and Lubricants” [39]. Elemental analyses data were obtained on a Thermo Quest Italia S.P.A. EA 1110 instrument.

EA 1110 equipment consists of the following: basic instrument, universal auto-sampler for both liquid or (and) solid samples, Sartorius XM 1000P electronic microbalance (SARTORIUS AG Göttingen, Germany) with bidirectional interface, computerized unit for controlling, running, data acquisition and processing, carrier gas cylinders (helium) and pure oxygen and respectively a gas cylinder for the pneumatic drive.

The process principle for determining the carbon content of the sample is based on three sequential steps: the sample, retained in a light thin capsule, is vigorously oxidized, resulting in a gaseous mixture that undergoes separation in a chromatographic column from which pure flue gases eluted are further on passed through a thermal conductivity detector (TCD) that generates an electrical signal proportional to the amount of eluted gas. The process known as Dynamic Flash Combustion is the method that ensures these conditions.

The four components resulting from the fuel blend are eluted and separated on a Porapack PQS column and then detected by the means of a TCD detector according to the following sequence N_2_, CO_2_, H_2_O, SO_2_. The temperature of the combustion furnace is maintained at 1000 °C, while during the combustion of the sample, the temperature attains 1800 °C. The measuring range for C, H, S, N, and O is within the limits: 0.01–100%. The results in percentage for each component are displayed by the equipment software.

## 3. Experimental Results for Fuel Blends’ Physical–Chemical Properties

Measured values for all samples are tabulated and centralized in Table 1.

It should be mentioned that Low Calorific Power (LCP) and Elemental analysis (EA) were determined only for Ke, SF, and SFP, while for the tested fuel blends they were computed according to reference [40].

Analyzing the data tabulated in Table 1, the following conclusions can be drawn:

- SFP oil has a flash point that is significantly higher than SF oil, while the flash point of both types of biodiesel is higher than Ke.
- The kinematic viscosity at 40 °C of both types of biodiesel used for blending are very close to each other but higher than that of Ke.
- The density of SF is slightly higher than that of SFP, but both types of biodiesel used for blending exhibit higher densities than Ke.
- The freezing point of SF is lower than the one of SFP, but significantly higher than that of Ke.
- LCP of SFP is higher than that of SF but each of them possess a LCP lower than Ke.
- Regarding the ES of the two types of biodiesel used for blending with Ke, it can be concluded that the percentage of the carbon content is lower for Ke, while its oxygen percentage is higher.
- For all analyzed fuel blends, the proportionality is maintained. The kinematic viscosity at 40 °C, the flash point, the density, and freezing point of the tested fuel blends increase along with the increase of the biodiesel percentage in the fuel blends, while LCP decreases along with the increase of the biodiesel percentage in the fuel blends. Regarding EA of the tested fuel blends, it can be concluded that the increase of the biodiesel percentage in the fuel blends produces an increase in the percentage of the oxygen content while the percentage of the carbon content decreases.

**Figure 1:**
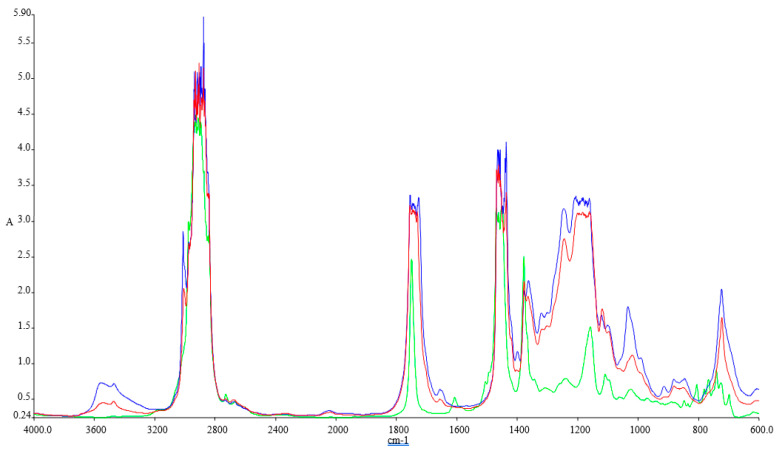
FTIR spectra of Green-Ke, Blue-SF, and Red-SFP.

**Figure 2:**
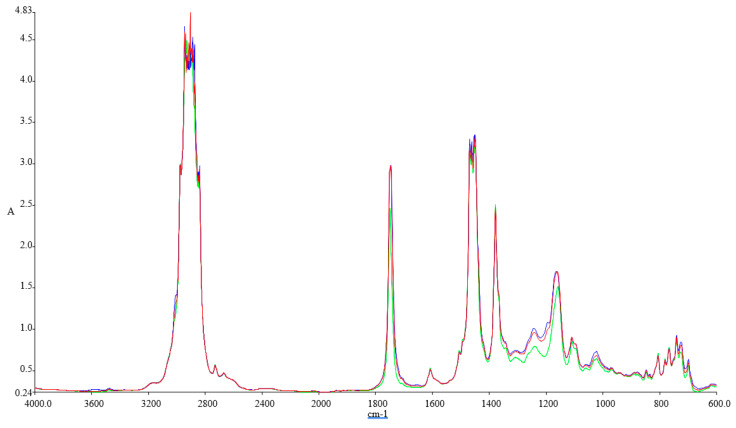
FTIR spectra of the tested fuel blends: Green-Ke, Blue-Ke+10%SF, and Red–Ke+10%SFP.

**Figure 3:**
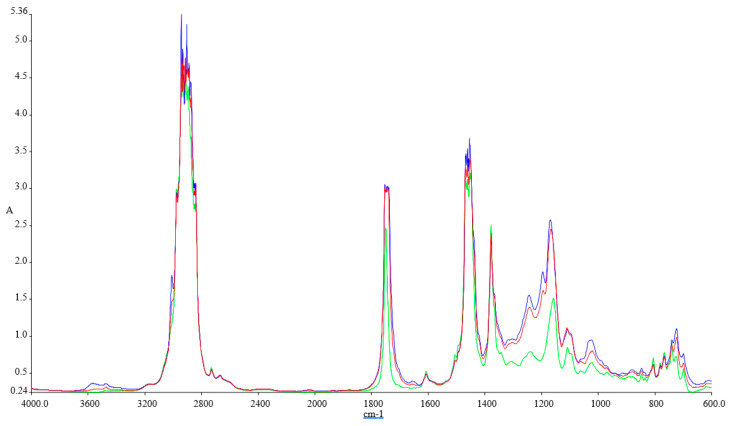
FTIR spectra of the tested fuel blends: Green-Ke, Blue-Ke+30%SF, and Red–Ke+30%SFP.

**Figure 4:**
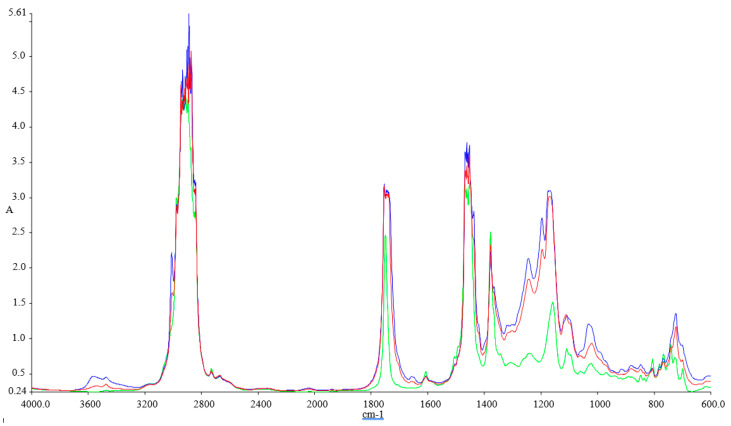
FTIR spectra of the tested fuel blends: Green-Ke, Blue-Ke+50%SF, and Red–Ke+50%SFP.

Figure 1, Figure 2, Figure 3 and Figure 4 illustrate the FTIR results.

The FTIR spectra inspected for the blends show variations at 1745.83 cm^−1^ (C=O stretching), 1030.98 cm^−1^, 1117.54 cm^−1^, and 1170.23 cm^−1^ (C–O alkoxyl stretching), they are visible in these blends, but their intensities vary according to the concentration of the biodiesel. These peaks increase with the concentration of biodiesel present in each of the blend, and this shows that the fatty acid methyl esters (FAME) is an indication of the amount of the biodiesel present in each of the biodiesel blend with kerosene, since FAME exhibits its appearance at 1745.83 cm^−1^ and 1170.23–1030.98 cm^−1^.

Methyl esters also show their absorptions characteristics in the peak around 1820–1680 cm^−1^, which is typical for carbonyl absorption. We also discovered variations in the intensities within the region of 678.55–721.41 cm^−1^ (=C–H bending; cis–di-substituted alkenes and aromatic). Their intensities were also found to increase with biodiesel concentration in each of the spectrum obtained [41].

## 4. Test Bench Experiment

The turbo engine’s test bench, methods, equipment, and testing procedure are presented.

**Figure 5:**
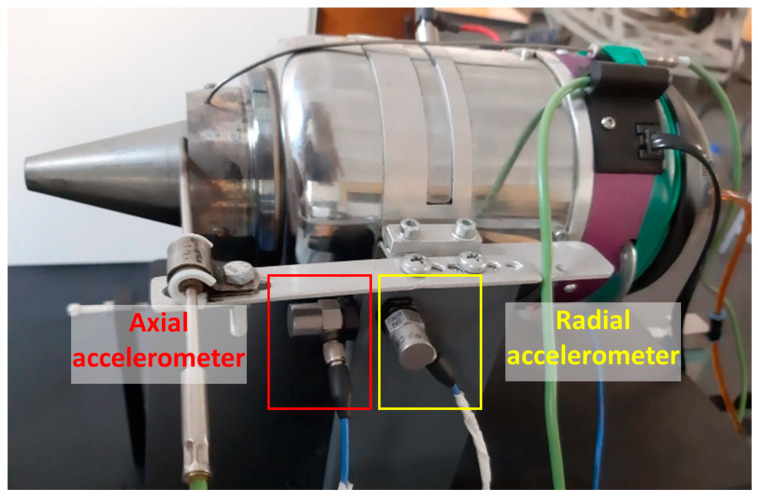
Test bench instrumentation and accelerometers location.

The experiments were performed on a Jet CAT P80^®^ micro-turbo engine [42], as shown in Figure 5, which is deployed at the Turbo Engines Laboratory of the Aerospace Engineering Faculty, “Polytechnic” University of Bucharest.

The studied fuel blends have been detailed in Section 2 and consisted of nine different liquids. It is to be mentioned that 5% Aeroshell 500 oil was added for engine lubrication.

Tests have been performed using different operating regimes: idle (18.7% of maximum throttle), cruise (30%), intermediate (60%), and maximum (94% for safety conditions). For each of the above-mentioned regimes, a 1 min testing interval was selected during which the engine parameters have been monitored.

The turbo engine instrumentation recorded: temperature T_comp after the compressor and T_comb before the turbine, fuel consumption Qc, air flow, pressure in the combustion chamber, and force F.

The engine works at a constant speed of the shaft, which was not modified by the fuel blends, but in order to maintain the shaft speed, the fuel blends will be fed in the combustion chamber at various rates.

Taking into account the constant speed of the compressor when working with fuel blends results in the same pressure after the compressor and the same air flow being generated.

Several parameters such as the consumed fuel flow (Qc), the temperature in front of the turbine(T_comb), and thrust (F) can be comparatively assessed when the shaft speed is kept constant.

Two accelerometers have been installed on the turbo engine’s support and are recording the radial and axial vibrations based on which the stability of the turbo engine while functioning was evaluated. The measurements for vibration assessment were performed by using a Sirius multichannel acquisition system from Dewesoft, Trbovlje, Slovenia and two 352C03 accelerometers from PCB Piezotronics, New York, NY, USA.

## 5. Turbo Engine Experimental Results

The main parameters that were recorded during micro-turbo engine operation are presented in Table 2. For each regime, the data were averaged for 1 min and are as follows: thrust, fuel flow, gas temperature in front of the turbine, and axial and radial vibrations.

The first conclusion that occurs after scrolling through Table 2 is that the functionality and integrity of the engine were neither compromised nor endangered.

**Figure 6:**
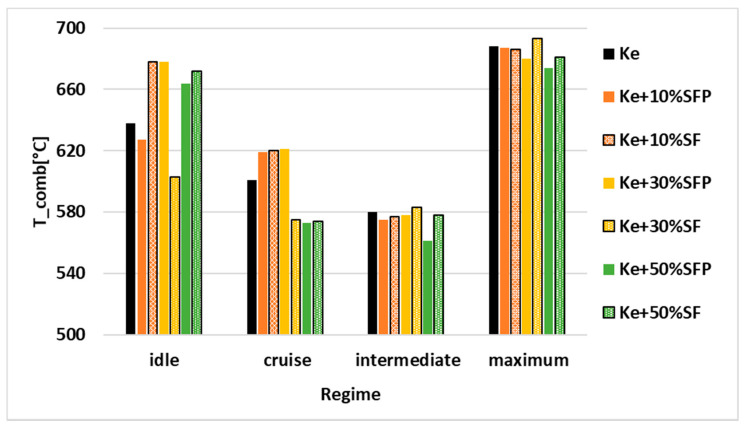
Variation of (T_comb) (°C) depending on regime and blends.

**Figure 7:**
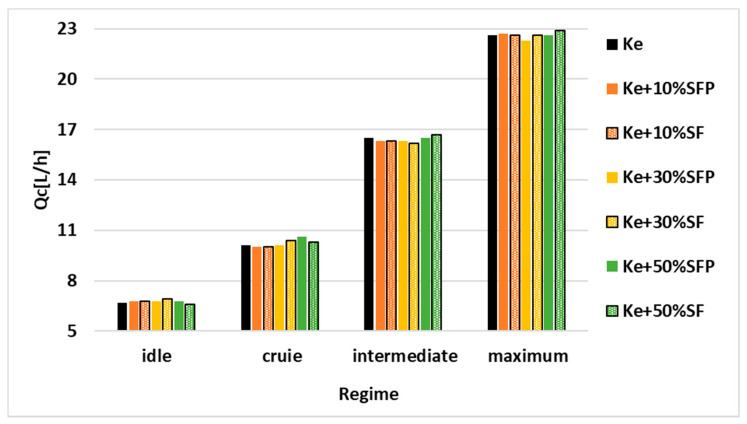
Variation of Qc (L/h) depending on the regime and blends.

**Figure 8:**
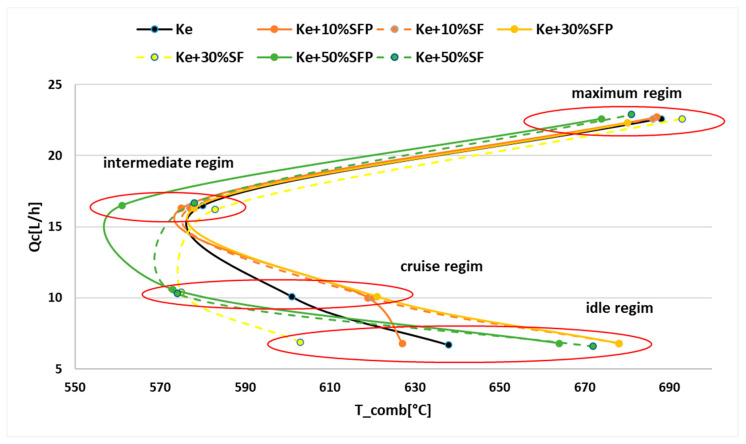
Correlation graph between Qc and T_comb.

**Figure 9:**
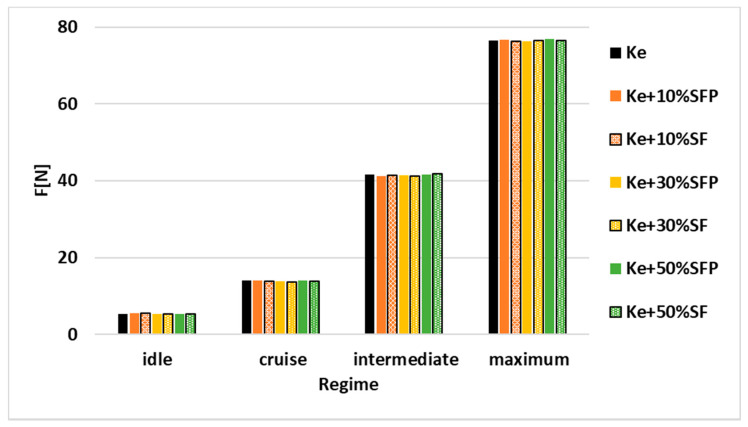
Variation of thrust F[N] depending on the regime and tested blends.

**Figure 10:**
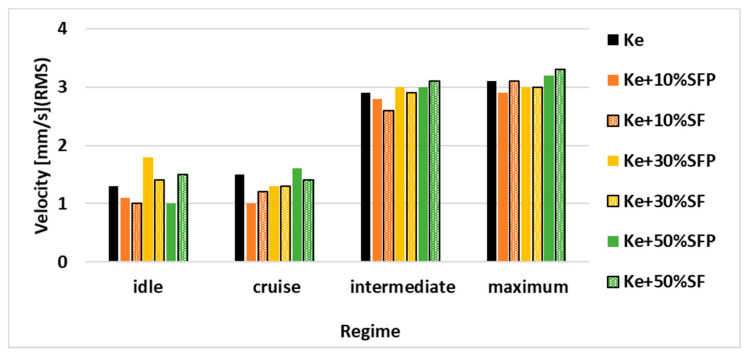
Radial vibration levels.

**Figure 11:**
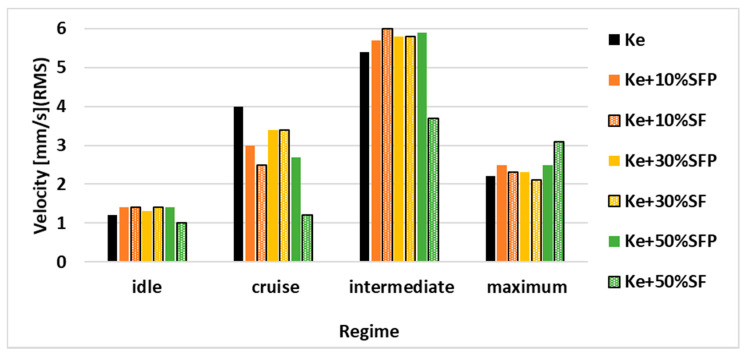
Axial vibration levels.

For a better visualization, the results shown in Table 2 are graphically represented in the following Figure 6, Figure 7, Figure 8, Figure 9, Figure 10 and Figure 11.

Figure 6 graphically illustrates the temperature variation in front of the turbine for all four operating regimes and all tested fuel blends.

Analyzing Figure 6, we can conclude that the engine was neither endangered nor compromised because the maximum temperature at which the turbine withstands, that is 800°C, was not even attained or exceeded.

The temperature oscillates from one fuel blend to another in idle regime, this variation being produced because the idle regime is considered an quite unstable one.

It can be observed that in the upper regimes intermediate respectively maximum the combustion temperatures tend to decrease along while the biodiesel concentration in the tested fuel blends increases.

Another observation that occurs is that in the case of fuel blend containing Ke + 50% SF, the combustion temperature is higher than in the case of fuel blend containing Ke + 50% SFP in all working regimes.

For the fuel blends with a concentration of 10% biodiesel, higher temperatures are obtained in the case of testing the fuel blends SF than in the case of testing the fuel blends containing SFP.

As for the fuel blends with a concentration of 30% biodiesel, the combustion temperature is higher in the following cases: (a) when the micro-turbo engine works in the low regimes, idle respectively cruise, being powered by a fuel blend containing SFP; (b) when the micro-turbo engine works in the upper regimes, intermediate respectively maximum respectively, and being powered by a fuel blend containing SF.

All these variations of the combustion temperature should be regarded because of the physical–chemical properties of the analyzed biodiesels. The variation of the temperature in correlation with other parameters will be reflected in both the specific fuel consumption and combustion yield into the combustion chamber.

In Figure 7, the variation of the consumed fuel flow expressed in liters/hour is illustrated.

It can be observed that there are no significant variations of the fuel flow neither for any of the four working regimes nor for any of the tested fuel blends. However, a slight increase in the consumed fuel flow may be noticed as biodiesel concentration in the tested fuel blends increases.

Analyzing both Figure 6 and Figure 7, it can be concluded that the variation of the consumed fuel flow (Qc) can be correlated with the variation of combustion temperature T_comb according to Figure 8. As it can be observed, a lower combustion temperature is corresponding to an increase of the consumed fuel flow.

Variation of thrust F depending on the regime and tested blends is illustrated in Figure 9.

It can be observed that the thrust has a slight upward trend in all the four regimes when biodiesel concentration in the tested fuel blends increases. This increase may be due to the variation of the fuel flow observed in Figure 7, knowing that as the determinations of the densities demonstrates, the blends have higher densities than Ke.

Analyzing the vibration results, presented in Figure 11 it can be observed that there are no significant variations of these levels, reported to the use of Ke considered as reference, that could endanger the functional integrity of the engine.

## 6. Jet Engine Performance Analysis

The performance parameters are calculated according to [43].

Determination of the density for each tested fuel blend allowed accomplishing the transformation of the fuel flow, which is measured by the means of the engine instrumentation, from liters per hour to kg per second. The specific consumption S is defined in Equation (1):

(1) $$S=3600\cdot \frac{\dot{M}c}{F}\left[\frac{\mathrm{kg}}{N\cdot h}\right]$$

where $\dot{M}c$ represents the fuel flow in kg/s.

As it concerns both the analysis of the development of the combustion into the combustion chamber and the estimation of combustion completeness, in order to determine the value of the combustion efficiency η_b_, this is expressed as follows (Equation (2)):

(2) $$\eta_{b}=\frac{\left(\dot{M}c+\dot{M}a\right){\mathrm{cp}}_{\_\mathrm{comb}}\cdot T_{\_\mathrm{comb}}-\dot{M}a\cdot {\mathrm{cp}}_{\_\mathrm{comp}}\cdot T_{\_\mathrm{comp}}}{\dot{M}c\cdot \mathrm{LCP}}$$

where LCP—Lower Calorific Power, cp—specific heat capacity, T_comp—temperature in front of the combustion chamber (that was recorded). Table 3 presents the calculated performances.

**Figure 12:**
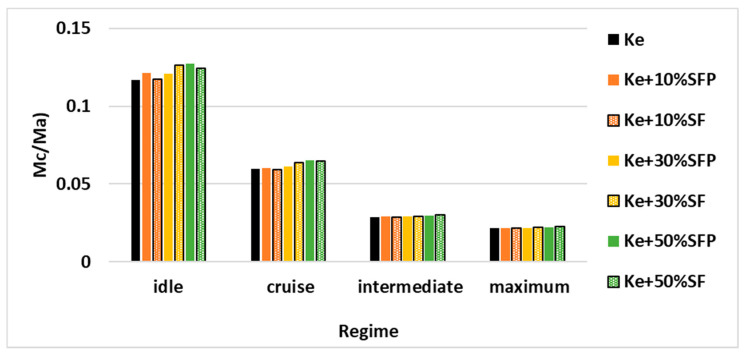
Variation of $\dot{M}c$/$\dot{M}a$ for all four regimes and for all the tested fuel blends.

**Figure 13:**
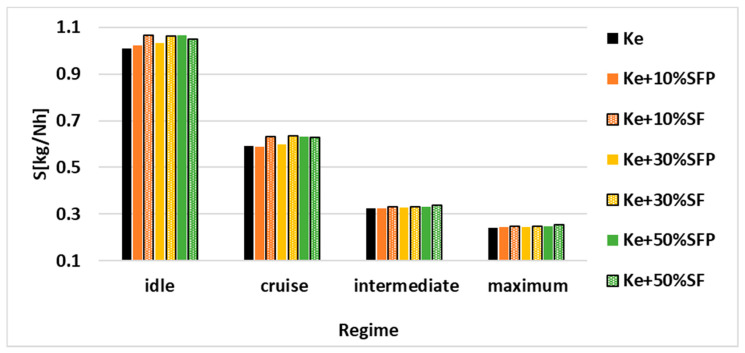
Variation of specific consumption for all four regimes and for all the tested fuel blends.

**Figure 14:**
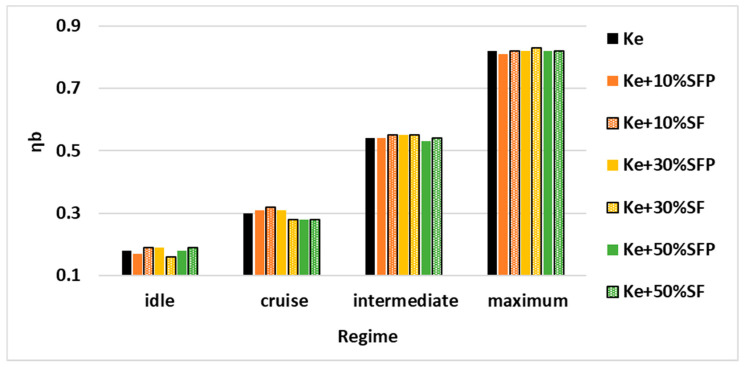
Variation of combustion efficiency for all the four regimes and for all the tested fuel blends.

For better visualizing the variation of the parameters systemized in Table 3, these were transposed into the graphical representations illustrated in Figure 12, Figure 13 and Figure 14.

Figure 12 illustrates the variation of the ratio between the fuel flow and the air flow passing through the combustion chamber.

There may be observed a slight increase of this ratio as the biodiesel concentration in the tested fuel blends increases. In addition, this ratio exhibits a more prominent increase, when the micro-turbo engine works in regimes idle respectively cruise, as the biodiesel concentration in the tested fuel blends increases.

Figure 13 illustrates the variation of the specific consumption, the most important parameter that allows the establishing of the micro-turbo engine performance, as it encompasses both the thrust of the engine and fuel consumption according to Equation (1).

An increase of specific consumption for each operating regime can be clearly observed as the biodiesel concentration in the tested fuel blends increases. Moreover, it can be seen that fuel blends comprised of SF have a higher specific consumption than those comprised of SFP.

Figure 14 illustrates the variation of the combustion efficiency for all four operating regimes and all tested fuel blends.

As can be observed, the efficiency of the combustion does not vary significantly in the upper working regimes of the micro-turbo engine, which proves the stability of the combustion for all tested fuel blends. However, in the lower working regimes of the micro-turbo engine, variations in the combustion efficiency can be observed for the same working regime when using different fuel blends.

Analyzing Figure 12 and respectively Figure 13, it can be concluded for working regime cruise that in those tests where a higher combustion efficiency is achieved, a lower specific consumption for the tested fuels is also observed.

## 7. Conclusions

The measurements made on the Jet CAT P80^®^ micro-turbo engine are showing that the addition of biodiesel in classical fuel does not endanger the functionality of the turbo engines.

Following the determinations, it was found that SF has a lower freezing point than SFP, giving it an advantage, but a higher concentration of biodiesel in the fuel blends will increase the freezing point, making them improper for use at high altitudes without being heated.

The calorific power of SF is slightly lower than that of SFP resulting in both a decrease of the calorific power when the biodiesel concentration in the fuel blends increases and an increase of the specific consumption of the fuel blends.

In terms of percentages of carbon content in the molecule, both biodiesels, used for blending with Ke in order to obtain the tested fuel blends, have a lower percentage than Ke, which results in a smaller amount of CO_2_ when are burned. Moreover, biodiesels (SF and SFP) have a greater amount of oxygen in the molecule than kerosene, and this is why they require lower amounts of air for combustion. This will result in the generation of a lower amount of NO_x_ after combustion compared to Ke. So, the slightly lower combustion temperatures of the fuel blends, consisting of biodiesel (SF/SFP) and Ke, can be explained by the fact that the oxygen in the molecule of biodiesel is used in combustion, and the use of air in excess is not required as in the case of Ke alone.

If considering the combustion products, SF is more advantageous for blending with Ke because of both the lower percentage of carbon content and the higher percentage of oxygen in the molecule than SFP. This proportion is also observed for the tested fuel blends.

In terms of engine performance, fuel specific consumption increases along with the increasing of the biodiesel percentage in the tested blends. The tests performed during the experimental campaign highlighted that the specific consumption of the fuel blends containing SF is lower than that of the fuel blends containing SFP, this being also attributed to their calorific powers.

As for the vibrations, they did not endanger the integrity of the engine.

In accordance with the mentioned conclusions, the research results can be applied to micro turbo-engines flying at lower altitudes. The future work will focus on the operation of a bigger turbo-engine with the assessment of the main parameters, which will allow us to determine the engine performance and the achievement of the main emissions consisting of green house gases.

## Figures and Tables

**Table 1:** Experimental results of the physical–chemical properties for fuel blends.

Sample	Flash Point °C	Kinematic Viscosity at 40 °C cSt	Density at 22 °C g/cm^3^	Freezing Point °C	Low Calorific Power kJ/kg	Elemental Analysis
Ke	42.3	1.39	0.817	<−35 °C	42,399	C% = 85.17 H% = 13.31 N% = 0.07 O% = 1.45
SF	86	5	0.884	−9 °C	36,956	C% = 77.28 H% = 12.00 N% = 0.07 O% = 10.65
SFP	161	5.08	0.875	−6 °C	38,300	C% = 78.65 H% = 12.61 N% = 0.07 O% = 8.67
Ke+ 10% SF	42.9	1.55	0.824	<−35 °C	41,855	C% = 84.38 H% = 13.18 N% = 0.07 O% = 2.37
Ke+ 30% SF	49.7	1.98	0.839	<−35 °C	40,766	C% = 82.8 H% = 12.92 N% = 0.07 O% = 4.21
Ke+ 50% SF	53.5	2.54	0.854	−25 °C	39,678	C% = 81.23 H% = 12.65 N% = 0.07 O% = 6.05
Ke+ 10% SFP	45.6	1.75	0.832	<−35 °C	41,989	C% = 84.52 H% = 13.24 N% = 0.07 O% = 2.17
Ke+ 30% SFP	53.5	2.54	0.854	−29 °C	41,169	C% = 83.21 H% = 13.1 N% = 0.07 O% = 3.62
Ke+ 50% SFP	71	3.37	0.853	−23 °C	40,350	C% = 81.91 H% = 12.96 N% = 0.07 O% = 5.06

**Table 2:** Micro-turbo engine parameters.

Blend	Regime	(T_Comb) (°C)	Qc (L/h)	F (N)	Acc-Axial (mm/s)	Acc-Radial (mm/s)
Ke	idle −18.70%	638	6.7	5.4	1.2	1.3
	cruise −30%	601	10.1	14.0	4	1.5
	intermediate −60%	580	16.5	41.6	5.4	2.9
	maximum −94%	688	22.6	76.4	2.2	3.1
(Ke)+ 10% SF	idle −18.70%	678	6.8	5.6	1.4	1
	cruise −30%	620	10.0	13.9	2.5	1.2
	intermediate −60%	577	16.3	41.4	6	2.6
	maximum −94%	686	22.6	76.2	2.3	3.1
(Ke)+ 30% SF	idle −18.70%	603	6.9	5.4	1.4	1.4
	cruise −30%	575	10.4	13.7	3.4	1.3
	intermediate −60%	583	16.2	41.2	5.8	2.9
	maximum −94%	693	22.6	76.5	2.1	3
(Ke)+ 50% SF	idle −18.70%	672	6.6	5.4	1	1.5
	cruise −30%	574	10.3	13.9	1.2	1.4
	intermediate −60%	578	16.7	41.9	3.7	3.1
	maximum −94%	681	22.9	76.5	3.1	3.3
(Ke)+ 10% SFP	idle −18.70%	627	6.8	5.5	1.4	1.1
	cruise −30%	619	10.0	14.0	3	1
	60%	575	16.3	41.3	5.7	2.8
	maximum −94%	687	22.7	76.7	2.5	2.9
(Ke)+ 30% SFP	idle −18.70%	678	6.8	5.4	1.3	1.8
	cruise −30%	621	10.1	13.9	3.4	1.3
	intermediate −60%	578	16.3	41.4	5.8	3
	maximum −94%	680	22.3	76.2	2.3	3
(Ke)+ 50% SFP	idle −18.70%	664	6.8	5.3	1.4	1
	cruise −30%	573	10.6	14.1	2.7	1.6
	intermediate −60%	561	16.5	41.6	5.9	3
	maximum −94%	674	22.6	76.8	2.5	3.2

**Table 3:** Calculated performances obtained for all four regimes and for all tested blends.

Blend	Regime	$\dot{M}c$/$\dot{M}a$	S (kg/Nh)	ηb
Ke	idle −18.70%	0.1166	1.008	0.18
	cruise −30%	0.0595	0.590	0.30
	intermediate −60%	0.0288	0.324	0.54
	maximum −94%	0.0215	0.242	0.82
(Ke)+ 10% SF	idle −18.70%	0.1171	1.000	0.19
	cruise −30%	0.0591	0.588	0.32
	intermediate −60%	0.0287	0.325	0.55
	maximum −94%	0.0217	0.244	0.82
(Ke)+ 30% SF	idle −18.70%	0.1261	1.063	0.16
	cruise −30%	0.0638	0.634	0.28
	intermediate −60%	0.0294	0.330	0.56
	maximum −94%	0.0221	0.248	0.83
(Ke)+ 50% SF	idle −18.70%	0.1245	1.050	0.19
	cruise −30%	0.0646	0.628	0.28
	intermediate −60%	0.0304	0.340	0.55
	maximum −94%	0.0227	0.256	0.82
(Ke)+ 10% SFP	idle −18.70%	0.1211	1.022	0.17
	cruise −30%	0.0600	0.588	0.31
	intermediate −60%	0.0290	0.325	0.54
	maximum −94%	0.0218	0.243	0.81
(Ke)+ 30% SFP	idle −18.70%	0.1208	1.031	0.19
	cruise −30%	0.0611	0.599	0.31
	intermediate −60%	0.0293	0.327	0.55
	maximum −94%	0.0218	0.243	0.82
(Ke)+ 50% SFP	idle −18.70%	0.1272	1.065	0.18
	cruise −30%	0.0653	0.631	0.28
	intermediate −60%	0.0298	0.333	0.53
	maximum −94%	0.0221	0.247	0.82

## Data Availability

The datasets used and analyzed during the current study are available from the corresponding author on request.
